# Impact on respiratory tract infections of heptavalent pneumococcal conjugate vaccine administered at 3, 5 and 11 months of age

**DOI:** 10.1186/1465-9921-8-12

**Published:** 2007-02-21

**Authors:** Susanna Esposito, Alessandro Lizioli, Annalisa Lastrico, Enrica Begliatti, Alessandro Rognoni, Claudia Tagliabue, Laura Cesati, Vittorio Carreri, Nicola Principi

**Affiliations:** 1 Institute of Pediatrics, University of Milan, Fondazione IRCCS "Ospedale Maggiore Policlinico, Mangiagalli e Regina Elena", Milan, Italy; 2 Department of Health Sciences, Regione Lombardia, Milan, Italy

## Abstract

**Background:**

Medical and public health importance of pneumococcal infections justifies the implementation of measures capable of reducing their incidence and severity, and explains why the recently marketed heptavalent pneumococcal conjugate vaccine (PCV-7) has been widely studied by pediatricians. This study was designed to evaluate the impact of PCV-7 administered at 3, 5 and 11 months of age on respiratory tract infections in very young children.

**Methods:**

A total of 1,571 healthy infants (910 males) aged 75–105 days (median 82 days) were enrolled in this prospective cohort trial to receive a hexavalent vaccine (DTaP/IPV/HBV/Hib) and PCV-7 (n = 819) or the hexavalent vaccine alone (n = 752) at 3, 5 and 11 months of age. Morbidity was recorded for the 24 months following the second dose by monthly telephone interviews conducted by investigators blinded to the study treatment assignment using standardised questionnaires. During these interviews, the caregivers and the children's pediatricians were questioned about illnesses and the use of antibiotics since the previous telephone call. All of the data were analysed using SAS Windows v.12.

**Results:**

Among the 1,555 subjects (98.9%) who completed the study, analysis of the data by the periods of follow-up demonstrated that radiologically confirmed community-acquired pneumonia (CAP) was significantly less frequent in the PCV-7 group during the follow-up as a whole and during the last period of follow-up. Moreover, there were statistically significant between-group differences in the incidence of acute otitis media (AOM) in each half-year period of follow-up except the first, with significantly lower number of episodes in children receiving PCV-7 than in controls. Furthermore, the antibiotic prescription data showed that the probability of receiving an antibiotic course was significantly lower in the PCV-7 group than in the control group.

**Conclusion:**

Our findings show the effectiveness of the simplified PCV-7 schedule (three doses administered at 3, 5 and 11–12 months of age) in the prevention of CAP and AOM, diseases in which *Streptococcus pneumoniae *plays a major etiological role. A further benefit is that the use of PCV-7 reduces the number of antibiotic prescriptions. All of these advantages may also be important from an economic point of view.

## Background

Respiratory tract infections are the most common diseases of infants and children, and have a significant impact on the patients themselves, their families and society as a whole [1-3]. Particularly in younger patients, they are mainly due to viruses, but bacteria also may play a significant causative role [4,5].

The most quantitatively and qualitatively important bacterial pathogen is *Streptococcus pneumoniae*, which is the main cause of mild but very common diseases, such as acute otitis media (AOM) and rhinosinusitis, as well as quite rare but potentially severe illnesses, such as community-acquired pneumonia (CAP) and meningitis [6-8]. Medical and public health importance of pneumococcal infections justifies the implementation of measures capable of reducing their incidence and severity, and explains why the recently marketed heptavalent pneumococcal conjugate vaccine (PCV-7) has been widely studied by pediatricians [9-14]. However, all of the currently available data concerning the clinical impact of PCV-7 on infants and children have been collected in subjects receiving four vaccine doses in accordance with the schedule usually used in the United States and many other industrialised countries: the administration of three doses at 2, 4 and 6 months of age, and a booster dose at 12–15 months [15-20].

It has recently been demonstrated that a simplified administration schedule based on two PCV-7 doses at 3 and 5 months of age, and a booster dose at 11–12 months, can be as immunogenic as the traditional four-dose schedule in both premature and full-term infants [21,22]. It has also been shown that the geometric mean antibody titres against all of the *S. pneumoniae *serotypes included in PCV-7 after the second and the third dose of the simplified scheme are quite similar to those found in children after the third and fourth dose of the four-dose regimen regardless of gestational age [21,22].

The administration of this simplified schedule, which is routinely used for all childhood vaccinations in some European countries (such as Italy and the Scandinavian countries) may reduce the costs of PCV-7, as well as any problems related to its supply and administration. However, as it is important to demonstrate its effectiveness in clinical practice, this study was designed to evaluate the impact of PCV-7 administered at 3, 5 and 11 months of age on respiratory tract infections in very young children.

## Methods

### Study design

This multicentre, prospective, observational, single-blind study was conducted in Italy in accordance with the principles of the Declaration of Helsinki.

Some of the vaccines recommended for infants and children worldwide (polio, diphtheria, tetanus, HBV) are mandatory by law in Italy, and administered in specific public centres of the Department of Health Sciences in each Region. Approximately 30 days after the birth of a child, the family receives an invitation to take the baby to the nearest vaccination centre between the 60^th ^and 90^th ^day of life in order to receive the first dose of the mandatory vaccines, and plan the administration of the other doses. On the same occasion, the other recommended vaccines (i.e., pertussis, *Haemophilus influenzae *type b, PCV-7, and conjugate meningococcal vaccine) are offered to the parents, who are free to decide which vaccines can be simultaneously administered to their child. Taking this usual behaviour into account, and together with the health workers of the vaccination centres in Lombardy, we decided to evaluate the possible impact of PCV-7 on infants and children when the vaccine was first marketed in Europe. Risks and benefits of PCV-7 were fully explained to the parents and they could accept or refuse the vaccination of their child. As all of the vaccines usually administered to Italian children are given using simplified schedules that foresee two doses in the first half year of life (at 3 and 5 months of age) and a booster dose at about one year (11–12 months), we decided to offer parents with no payment the administration of PCV-7 during the visits planned for the other vaccines.

The study protocol was approved by the Ethics Committee of the University of Milan, and all of the caregivers gave their written informed consent before enrolment. Guidelines for human experimentation were followed in the conduct of clinical research. A single-blind design was chosen because the preparation of a placebo containing all of the components of the formulation except the pneumococcal antigens was technically impossible.

### Study population and vaccine usage

Between 1 September and 31 December 2002, all of the healthy children presenting at 11 vaccination centres in Lombardy were considered for enrolment. The children with known immunodeficiency, any serious chronic or progressive disease, or a history of seizures were excluded, as were those born to HbsAg- or HCV-positive mothers, those with a known allergy to any of the vaccine components, those who had received a treatment likely to alter the immune response (i.e. intravenous immunoglobulin, blood products, systemic steroids for more than two weeks, anticancer therapy) in the previous four weeks, those who had received antipyretic and/or analgesic drugs in the four hours before vaccine administration, and those with a history of pneumococcal disease. Moreover, at the time of immunisation, all of the study subjects had to be in good physical condition.

After giving information regarding the characteristics, efficacy and side effects of the mandatory and recommended vaccines (including PCV-7), the first doses of both were given. The parents could choose to administer or not to their children PCV-7. In order to make the group of study children as homogeneous as possible, only the subjects receiving a combined hexavalent formulation (DTaP/IPV/HBV/Hib) containing pertussis and *H. influenzae *type b vaccines together with the four mandatory vaccines were considered and divided into two groups: the first consisted of those whose parents decided to administer to their children both the hexavalent vaccine and PCV-7 (PCV-7 group), and the second of those receiving only the hexavalent vaccine (control group). Parents that did not choose PCV-7, whose children represented the control group, justified their decision explaining that at the time of the study few data were available on the effectiveness of PCV-7 (n = 376), no data were available on the effectiveness of the 3-dose schedule (n = 273) and the fear of side effects (n = 103). No difference in refuse rate between the vaccination centres was observed. All of the enrolled children received the vaccinations at three, five (primary series) and 11 months of age (booster). Both vaccines were given intramuscularly in opposing thighs.

The enrolment was prospective. The vaccines were administered by the health workers of the 11 participating vaccination centres, who were supervised by two investigators (A.L. and V.C.); morbidity was monitored in a uniform way by five investigators of the University of Milan's Institute of Pediatrics, who were blinded to the study treatment assignment (A.L., E.B., A.R., C.T. and L.C.). The parents and caregivers were instructed not to inform the examiner whether their child had received PCV-7 or not.

All of the children received their first vaccine doses between 1 September and 31 December 2002; morbidity was recorded for the 24 months after the month following the second dose.

### Procedures

Before enrolment, each subject's medical history was reviewed in order to ensure compliance with the inclusion and exclusion criteria. Their demographic data and medical history were recorded at enrolment, and they underwent a physical examination including rectal temperature. After the administration of a single dose of vaccine, each subject was observed for a minimum of 15 minutes. Emergency management supplies (AMBU bag, adrenalin and antihistamines) were available for the initial treatment of an allergic reaction if needed. The parents or legal guardians were asked to record on the diary card any adverse events, unscheduled physician visits, and the use of concomitant prescription and non-prescription medication at any time during the study period, and to contact the investigator immediately if any significant illness or hospitalisation occurred.

During the surveillance of morbidity, information regarding illnesses and related morbidities among the study subjects was obtained in a uniform way by means of monthly telephone interviews conducted, by the same investigators blinded to the study treatment assignment, using standardised questionnaires [[23-25]; see Additional file 1]. During these interviews, the caregivers were questioned about illnesses and the use of antibiotics since the previous telephone call. The parents or legal guardians were asked to answer a list of questions regarding their child's disease: e.g., physician's final diagnosis, administered medication, hospitalisation, duration of signs/symptoms, medical visits, examinations, the number of lost day-care days. They were also asked to specifiy any change in the family's composition and income, the household's smoking habits, and the attendance at day-care centres, schools or work of the family members. For each episode of illness reported by the parents or legal guardians, a telephone call was made to the pediatrician responsible for the study child in order to confirm the diagnosis, the prescribed therapy and the final outcome. In presence of two different diseases at the same episode of illness, the most severe diagnosis was considered in the analysis; each episode of illness presented by the children was took into account as single event in the analysis. Pediatricians of the study children were also monthly queried in order to check for episodes of illness or symptoms that were forgotten by the parents. The episodes were defined on the basis of standard criteria [26] and divided into three main categories: 1) respiratory tract infections (RTIs), including both upper (i.e. rhinitis, pharyngitis, sinusitis) (URTIs) and lower respiratory tract infections (i.e. acute bronchitis, bronchiolitis, infectious wheezing, radiologically confirmed community-acquired pneumonia, CAP) (LRTIs); 2) acute otitis media (AOM); and 3) other illnesses not associated with respiratory tract infections or AOM. Antibiotic courses were considered as the total number of courses, and the number of courses for the categories of 1) URTIs; 2) LRTIs, including CAP; 3) AOM; and 4) other illnesses.

### Statistical analysis

All of the data were analysed by investigators blinded to treatment groups using SAS Windows v.12. The continuous variables are presented as median values and ranges, and the categorical variables as numbers and percentages with the relative risk (RR) and 95% confidence intervals (95% CI). All of the children were included in the comparisons of morbidity and antibiotic use between the PCV-7 and the control group. For the purposes of analysis, the incidence of respiratory diseases and the number of antibiotic courses were calculated for the follow-up period as a whole, and for each half-year of follow-up. If children had multiple episodes of illness, each event was considered and treated separately in the analysis. A *p*-value of < 0.05 was considered significant for all statistical tests. The parametric data were analysed using analysis of variance (PROC GLM and LSD options) with treatment terms. When the data were not normally distributed or were non-parametric, the Kruskal-Wallis test was used. The categorical data were analysed using contingency tables and the Chi-square or Fisher's test.

## Results

### Study population

The study involved 1,571 healthy infants (910 males) aged 75–105 days (median 82 days): 819 in the PCV-7 group and 752 in the control group. Eight children in the PCV-7 group and eight in the control group did not receive the three vaccination doses, and were therefore excluded from the clinical analysis. The final study group consequently included 1,555 subjects: 811 in the PCV-7 group and 744 in the control group. All these 1,555 children were contacted by monthly telephone interviews with no loss to follow-up. Considering the small dropouts and the fact that the intention to treat population showed similar characteristics and results to that observed in the final study group, only data of the children who completed the follow-up are presented.

Table 1 summarises the demographic characteristics of the children, and shows that the two groups were comparable, with no significant differences in terms of gender distribution, age at vaccination, or any of the variables usually considered risk factors for carrying respiratory pathogens, including *S. pneumoniae*. The distribution of children by center was the following: Milano, 416 (PCV-7 group, n = 214; control group, n = 202); Legnano, 216 (PCV-7 group, n = 110; control group, n = 106); Monza, 216 (PCV-7 group, n = 110; control group, n = 106); Cremona, 110 (PCV-7 group, n = 58; control group, n = 52); Pavia, 110 (PCV-7 group, n = 58; control group, n = 52); Mantova, 104 (PCV-7 group, n = 58; control group, n = 46); Melzo, 100 (PCV-7 group, n = 52; control group, n = 48); Brescia, 99 (PCV-7 group, n = 51; control group, n = 48); Breno, 67 (PCV-7 group, n = 36; control group, n = 31); Sondrio, 67 (PCV-7 group, n = 34; control group, n = 33); Varese, 50 (PCV-7 group, n = 27; control group, n = 23). Geographic areas of the different centres showed similar characteristics, with pneumococcal vaccination coverage less than 10% and *H. influenzae *vaccination coverage more than 90% during the study period.

**Table 1:** Demographic characteristics of the study children.

**Characteristics**	**PCV-7 group (n = 811)**	**Control group (n = 744)**	***P***
Males (%)	476 (58.7)	413 (55.5)	0.22
Age at vaccination, median days (range)			
First dose	82 (75–104)	82 (76–103)	0.96
Second dose	140 (128–155)	143 (130–158)	0.93
Third dose	340 (328–361)	343 (331–364)	0.92
Breast-feeding for at least three months, No. (%)	551 (67.9)	476 (64.1)	0.11
Living in urban area, No. (%)	527 (65.0)	498 (66.9)	0.44
Mother's age, median years (range)	33 (24–49)	33 (20–46)	0.96
Father's age, median years (range)	35 (21–50)	35 (21–51)	0.99
No. of persons in household, median (range)	3 (2–4)	3 (2–4)	1.00
No. of siblings, median (range)	1 (0–2)	1 (0–2)	1.00
Children with a smoker in the family, No. (%)	194 (23.9)	182 (24.5)	0.84
Children attending day-care, No. (%)	284 (35.0)	231 (31.0)	0.10
Duration of day care attendance, No. (%)			
0–6 months	79 (9.7)	65 (8.7)	0.55
7–12 months	33 (4.1)	44 (5.9)	0.11
13–18 months	111 (13.7)	94 (12.6)	0.59
19–24 months	78 (9.6)	58 (7.8)	0.23
Vaccinated against influenza, No. (%)	12 (1.5)	10 (1.3)	0.99

No significant between-group differences.

### Episodes of illness during the follow-up

In both groups, the most frequently reported illnesses were URTIs, followed by other illnesses, LRTIs and AOM. During the follow-up period, 3,877 episodes of respiratory tract infections were recorded in the children treated with PCV-7, and 3,460 in the control group, with no significant difference between the respective incidences of 239.0 and 232.5 episodes/100 child-years (RR: 1.02; 95% CI: 0.98–1.07; *p *= 0.32). However, analysis of the data by the periods of follow-up demonstrates that between the ages of 25 and 30 months of life, the children receiving PCV-7 suffered from a significantly lower rate of respiratory tract infections than the controls (RR: 0.90; 95% CI: 0.81–1.00; *p *= 0.047) (Table 2).

**Table 2:** Frequency of respiratory tract infections (RTIs) during follow-up.

**Episodes**	**PCV-7 group (n = 811)**	**Control group (n = 744)**	**RR**	**95% CI**	***P***
Total number of RTIs during follow-up	3,877	3,460			
Episodes/100 child-years	239.0	232.5	1.02	0.98–1.07	0.32
RTIs in children aged 6–12 months	1,035	928			
Episodes/100 child-years	255.2	249.4	1.02	0.94–1.12	0.62
RTIs in children aged 13–18 months	1,185	986			
Episodes/100 child-years	292.2	265.0	1.10	1.00–1.18	0.055
RTIs in children aged 19–24 months	998	872			
Episodes/100 child-years	246.1	234.4	1.05	0.96–1.15	0.31
RTIs in children aged 25–30 months	659	674			
Episodes/100 child-years	162.5	181.1	0.90	0.81–1.00	0.047

RR = relative risk; 95% CI = 95% confidence interval.

Regarding the URTI and LRTI data, the overall frequency of episodes during follow-up was similar in the PCV-7 and control group, with a mean number of episodes/100 child-years of respectively 194.6 and 190.3 URTIs (RR: 1.02; 95% CI: 0.87–1.05; *p *= 0.48), and 44.3 and 42.2 LRTIs (RR: 1.05; 95% CI: 0.94–1.27; *p *= 0.41). Although the analysis regarding the individual periods of follow-up does not demonstrate any significant between-group differences in terms of URTIs, there were 23% fewer LRTIs between the age of 25 and 30 months in the PCV-7 group than in the control group (RR: 0.77; 95% CI: 0.61–0.97; *p *= 0.023). In terms of the individual LRTI diagnoses, radiologically confirmed CAP was significantly less frequent in the PCV-7 group (RR: 0.35; 95% CI: 0.22–0.53; *p *< 0.0001) during the follow-up as a whole, and during the last period of follow-up (Table 3). All of the cases of CAP were radiologically confirmed and 33 subjects (61.1%) in the PCV-7 group and 106 (73.6%) in the control group (*p *< 0.0001) had been hospitalised.

**Table 3:** Frequency of radiologically confirmed community-acquired pneumonia (CAP) during follow-up.

**Episodes**	**PCV-7 group (n = 811)**	**Control group (n = 744)**	**RR**	**95% CI**	***P***
Total number of CAP cases during follow-up	27	72			
Episodes/100 child-years	1.7	4.8	0.35	0.22–0.53	<0.0001
CAP in children aged 6–12 months	9	7			
Episodes/100 child-years	2.2	1.88	1.17	0.44–3.16	0.74
CAP in children aged 13–18 months	3	9			
Episodes/100 child-years	0.7	2.4	0.30	0.08–1.11	0.07
CAP in children aged 19–24 months	7	16			
Episodes/100 child-years	1.72	4.30	0.40	0.16–0.97	0.04
CAP in children aged 25–30 months	8	40			
Episodes/100 child-years	1.97	10.7	0.18	0.09–0.39	<0.0001

RR = relative risk; 95% CI = 95% confidence interval.

Table 4 shows the frequency of AOM during follow-up. At least one episode of AOM was diagnosed in 478 children in the PCV-7 group (58.9%) and 499 in the control group (67.1%), for a mean number of episodes/100 child-years of respectively 637 and 698 (RR: 0.83; 95% CI: 0.61–1.02; *p *= 0.02). There were statistically significant between-group differences in the incidence of AOM in each half-year period of follow-up except the first. Recurrent AOM (defined as ≥ 3 episodes in six months or ≥ 4 in one year) [24] was reported in 29 children receiving PCV-7 (3.5%) and in 43 controls (5.8%) (RR: 0.62; 95% CI: 0.38–0.99; *p *= 0.044).

**Table 4:** Frequency of acute otitis media (AOM) during follow-up.

**Episodes**	**PCV-7 group (n = 811)**	**Control group (n = 744)**	**RR**	**95% CI**	***P***
Total number of AOM cases during follow-up	637	698			
Episodes/100 child-years	39.2	46.9	0.83	0.61–1.02	0.02
AOM in children aged 6–12 months	156	156			
Episodes/100 child-years	38.4	41.9	0.91	0.75–1.20	0.06
AOM in children aged 13–18 months	195	220			
Episodes/100 child-years	48.0	59,1	0.81	0.76–1.02	0.04
AOM in children aged 19–24 months	144	162			
Episodes/100 child-years	35.5	43.5	0.82	0.62–1.24	0.04
AOM in children aged 25–30 months	142	160			
Episodes/100 child-years	35.0	43.0	0.81	0.61–1.20	0.04

RR = relative risk; 95% CI = 95% confidence interval.

There were 2,562 other illnesses not associated with respiratory problems or AOM in the PCV-7 group, and 2,378 in the control group, for a mean number of episodes/100 child-years of respectively 157.9 and 159.8 (RR: 0.98; 95% CI: 0.93–1.04; *p *= 0.57). Among these other illnesses, there were 3 patients (0.4%) with invasive disease (two cases with sepsis due to *Neisseria meningitidis *and one with sepsis due to *Escherichia coli*) in the PCV-7 group and 5 (0.7%) with invasive disease in the control group (two cases with sepsis due to *S. pneumoniae*, two cases with sepsis due to *N. meningitidis *and one case with meningitis due to *S. pneumoniae*). There were no between-group differences in the rate of other illnesses during the individual periods of follow-up.

No difference in results was observed between the different centers.

### Antibiotic courses during follow-up

Table 5 shows the antibiotic prescriptions made during the follow-up. The total number of prescribed antibiotic courses was 2,020 in the PCV-7 group and 2,079 in the control group, for a mean number of courses/100 child-years of respectively 124 and 139 (RR: 0.89; 95% CI: 0.83–0.94; *p *= 0.0001). The antibiotic prescription data per period of follow-up show that the probability of receiving an antibiotic course was significantly lower in the PCV-7 group than in the control group, between the ages of 13 and 18 months (RR: 0.86; 95% CI: 0.76–0.95; *p *= 0.004), and between the ages of 25 and 30 months (RR: 0.78; 95% CI: 0.67–0.90; *p *= 0.0008). Results were similar when days of antibiotic therapy instead of antibiotic courses are considered.

**Table 5:** Antibiotic courses prescribed during follow-up.

**Episodes**	**PCV-7 group (n = 811)**	**Control group (n = 744)**	**RR**	**95% CI**	***P***
Total number of antibiotic courses during follow-up	2,020	2,076			
Courses/100 child-years	124	139	0.89	0.83–0.94	0.0001
Antibiotic courses in children aged 6–12 months	504	454			
Courses/100 child-years	122	120	1.01	0.89–1.14	0.87
Antibiotic courses in children aged 13–18 months	620	658			
Courses/100 child-years	153	177	0.86	0.76–0.95	0.004
Antibiotic courses in children aged 19–24 months	549	562			
Courses/100 child-years	135	151	0.89	0.79–1.01	0.06
Antibiotic courses in children aged 25–30 months	347	402			
Courses/100 child-years	85	108	0.78	0.67–0.90	0.0008

RR = relative risk; 95% CI = 95% confidence interval.

There were no significant between-group differences in antibiotic prescriptions when the individual disease categories were evaluated, with the exception of AOM. In this case, 25% fewer antibiotic courses were prescribed in the PCV-7 group (RR: 0.79; 95% CI: 0.71–0.94; *p *= 0.02).

No difference in results was observed between the different centers.

## Discussion

The results of this study suggest that the simplified PCV-7 schedule of only three doses administered at 3, 5 and 11–12 months of age can significantly reduce the incidence of AOM and CAP, as well as the consumption of antibiotics. These major advantages become apparent immediately after the administration of the third dose and are maintained until at least the end of the 30^th ^month of life. To the best of our knowledge, this is the first study which showed the impact of PCV-7 administered at 3, 5 and 11–12 months of age on respiratory tract infections. Previous studies have shown that the positive effects of the 4-dose PCV-7 schedule on respiratory tract infections can be seen in children aged more than 36 months [27,28] and, as both schedules lead to similar antibody levels [21,22], it is possible that the global prevention of pneumococcal respiratory diseases in children vaccinated with the simplified schedule is even greater than that demonstrated by our study.

In our study, a between-group difference in the incidence of RTIs and LRTIs was clearly evident only after the 25^th ^month of age. These findings may be explained by the fact that, as the bacterial/viral RTI ratio increases with age [5,29-33], the effect of pneumococcal vaccine appears more evident in children older than 2 years. On the basis of these results, one should consider the opportunity to give the vaccination as a single- or two-dose schedule in children older than one year of age. Although the effectiveness of these schedules has not been evaluated in this study, the vaccination in the first months of life permits to avoid or significantly reduce pneumococcal nasopharyngeal colonization with major benefits on respiratory morbidity [34,35].

Although the higher rates of CAP among older unvaccinated children have no specific explanation, the global incidence of CAP in our unvaccinated children of all age groups was similar to that found in previous studies [36,37]. However, the positive effect of PCV-7 on CAP prevention was greater than that previously reported with this vaccine [19] and similar to that observed with the 9-valent pneumococcal vaccine [38]. This was probably due to the fact that we considered only radiologically confirmed cases, the majority of which were severe enough to require hospitalisation.

The incidence of AOM in both of our groups fell within the previously reported range [39-41], but was significantly lower in the children receiving PVC-7 during the individual follow-up periods as well as during the follow-up as a whole. The reduction in AOM in our study population was greater than that found in clinical trials carried out in the United States [16,18,19] and Finland [17], which demonstrated that the efficacy of PCV-7 was 6–9% against all cases of AOM and 50–60% against cases due to the pneumococcal serotypes included in the vaccine. The global reduction in the incidence of AOM in our PCV-7 group was 17%, and the reductions in the individual 6-month periods were respectively 9%, 19%, 18% and 19%. These results are similar to those obtained with the 9-valent pneumococcal conjugate vaccine by Dagan *et al*. [28], who suggested that the greater-than-expected effect of the vaccine may have been due to it better coverage against antibiotic-resistant strains of *S. pneumoniae *[28], the large majority of which belong to a limited number of serotypes included in PCV-7 [34,42,43]. We do not know the incidence of antibiotic-resistant *S. pneumoniae *strains among our study children but, as recent data collected in Italy have demonstrated a significant increase in the prevalence of highly-resistant strains [44-46], the explanation offered by Dagan *et al*. [28] may also apply to our study.

The fact that the children in our PCV-7 group received significantly fewer antibiotic courses than those in the control group indicates that the vaccine may reduce not only the risk of adverse events due to antibiotic use, but also the costs of medical treatment. The economic saving is further underlined by the fact that the children in the PCV-7 group required fewer hospitalisations due to CAP.

Results of this study are in line with those calculated in children of the same areas regarding the predicted effects of PCV-7 immunization in relation to the circulation of the different *S. pneumoniae *serotypes and their role in nasopharyngeal colonization as well as in the determination of non-invasive diseases [32,43]. In addition, one of the major advantages of the 3-dose schedule is the fact that this simplified scheme could be associated with a reduction of 25% in health care costs in comparison with the traditional 4-dose schedule. Moreover, when high pneumococcal vaccination coverage levels are reached, further medical and economical benefits due to the effect of herd immunity could be observed.

Our findings may be criticised on the grounds that they come from a single-blind, observational study rather than a double-blind, randomised, placebo-controlled trial, but an analysis of the characteristics of the study children showed that there were no differences between the subjects receiving PCV-7 and the controls. In particular, there were no between-group differences in the variables that can influence the carrier state of respiratory pathogens and contribute to the development of respiratory diseases. Moreover, all of the information regarding the diseases was verified by means of telephone interviews with the children's pediatricians, and only the data reported by them were used in the analysis. Finally, the total number of diagnosed illnesses other than respiratory diseases or AOM in both of our study groups was similar, thus suggesting that there was no difference in the attention paid by the parents to the diseases developed by their children. On the basis of all of the above, we believe that there is only a marginal risk of sampling bias influencing our data analysis and that the two groups are therefore comparable.

## Conclusion

Our findings show the effectiveness of the simplified PCV-7 schedule (three doses administered at 3, 5 and 11–12 months of age) in the prevention of CAP and AOM, diseases in which *S. pneumoniae *plays a major etiological role. A further benefit is that the use of PCV-7 reduces the number antibiotic prescriptions. All of these advantages may also be important from an economic point of view.

## List of abbreviations

Acute otitis media, AOM

Community-acquired pneumonia, CAP

Confidence intervals, CI

Heptavalent pneumococcal conjugate vaccine, PCV-7

Lower respiratory tract infection, LRTI

Neisseria meningitidis, N. meningitidis

Relative risk, RR

Respiratory tract infection, RTI

Streptococcus pneumoniae, S. pneumoniae

Upper respiratory tract infection, URTI

## Competing interests

The author(s) declare that they have no competing interests.

## Authors' contributions

S.E. participated in the design as well as coordination of the study and helped to draft the manuscript; A.L. participated in the design of the study and performed the statistical analysis; A.L., E.B., A.R., C.T. and L.C. carried out the telephone interviews during the surveillance of morbidity; V.C. participated in the coordination of the study; N.P. conceived the study and wrote the manuscript.

## Supplementary Material

Additional File 1 Telephone survey used for surveillance of morbidity. The table shows the standardised questionnaire used for telephone interview during the surveillance of morbidity. Click here for file
