# Supplementary material for: Impact on respiratory tract infections of heptavalent pneumococcal conjugate vaccine administered at 3, 5 and 11 months of age
Source: Respir Res. 2007 Feb 21;8(1):12. doi: 10.1186/1465-9921-8-12 (PMC1804265; doi:10.1186/1465-9921-8-12)
Supplement: Additional File 1 — Telephone survey used for surveillance of morbidity. The table shows the standardised questionnaire used for telephone interview during the surveillance of morbidity. [file 1465-9921-8-12-S1.doc]

**Additional file 1: Telephone survey used for surveillance of morbidity.**

| **Question** | **Answer** |
| --- | --- |
| Investigator’s initials of name and surname | ____________________ |
| Contacted caregivers | ____________________ |
| Did the child present respiratory tract infections or acute otitis media since the previous telephone call? | NO |__| YES |__| *Specify number and type of episodes*   - Rhinitis, No. |__| - Sinusitis, No. |__| - Pharyngitis, No. |__| - Croup, No. |__| - Acute bronchitis, No. |__| - Infectious wheezing, No. |__| - CAP, No. |__| - Acute otitis media, No. |__| |
| Did the child present invasive diseases since the previous telephone call? | NO |__| YES |__| *Specify number and type of episodes:*   - Sepsis No. |__| - Meningitis No. |__| |
| How many episodes of fever did the child present since the previous telephone call? | ____________________ |
| Did the child present other medical problems since the previous telephone call? | NO |__| YES |__| *Specify type of medical problems______________*  *______________________________*  _______________________________ |
| How many medical visits did the child perform since the previous telephone call? | ____________________ |

**Table 1: Telephone survey (continue).**

| **Question** | **Answer** |
| --- | --- |
| Did the child perform diagnostic examinations since the previous telephone call? | NO |__| YES |__| *Specify type of exams __________________*  *____________________________*  ____________________________  ____________________________  ____________________________  ____________________________ |
| Has been the child hospitalized since the previous telephone call? | NO |__| YES |__| *Specify diagnosis and duration of hospitalization__________________*  *_____________________________*  _____________________________ |
| Did the child receive antibiotic therapy since the previous telephone call? | NO |__| YES |__| *Specify antibiotic, reason for therapy, dose and duration of therapy___________*  *_____________________________*  _____________________________  _____________________________  _____________________________  _____________________________ |
| Did the child receive other drugs since the previous telephone call? | NO |__| YES |__| *Specify drug, reason for therapy, dose and duration of therapy______________________*  *_____________________________*  _____________________________  _____________________________  _____________________________  _____________________________ |
| Did the child’s family show differences in demographic characteristics since the previous telephone call? | NO |__| YES |__| *Specify differences __________________*  *_____________________________*  _____________________________ |
